# Assessment of neutrophil subsets and immune checkpoint inhibitor expressions on T lymphocytes in liver transplantation: A preliminary study beyond the neutrophil-lymphocyte ratio

**DOI:** 10.3389/fphys.2023.1095723

**Published:** 2023-03-30

**Authors:** Arnaud Riff, Muzhda Haem Rahimi, Marie-Charlotte Delignette, Morgane Gossez, Rémy Coudereau, Solène Pantel, Teresa Antonini, François Villeret, Fabien Zoulim, Jean-Yves Mabrut, Jérome Dumortier, Fabienne Venet, Fanny Lebossé, Guillaume Monneret

**Affiliations:** ^1^ Hepatology Department, Hospices Civils of Lyon, Lyon Hepatology Institute, Croix-Rousse Hospital, Lyon, France; ^2^ Medical School, University of Lyon, Claude Bernard Lyon 1 University, Lyon, France; ^3^ Hospices Civils of Lyon, Immunology Laboratory, Edouard Herriot Hospital, Lyon, France; ^4^ Anaesthesiology and Critical Care Department, Hospices Civils of Lyon, Lyon Hepatology Institute, Croix-Rousse Hospital, Lyon, France; ^5^ Department of Digestive Surgery and Liver Transplantation, Hospices Civils of Lyon, Lyon Hepatology Institute, Croix-Rousse Hospital, Lyon, France; ^6^ Hepato-Gastroenterology Department, Hospices Civils of Lyon, Lyon Hepatology Institute, Edouard Herriot Hospital, Lyon, France; ^7^ Centre International de Recherche en Infectiologie (CIRI), INSERM U1111, CNRS, UMR5308, Ecole Normale Supérieure de Lyon, Université Claude Bernard-Lyon 1, Lyon, France

**Keywords:** transplantation, immunosuppression, cirrhosis, immune checkpoint receptors, PD-1, LOX-1

## Abstract

**Background:** Advanced stages of cirrhosis are characterized by the occurrence of progressive immune alterations known as CAID (Cirrhosis Associated Immune Dysfunction). In advanced cirrhosis, liver transplantation (LT) remains the only curative treatment. Sepsis, shares many similarities with decompensated cirrhosis in terms of immuno-inflammatory response. In both conditions, the neutrophil-lymphocyte ratio (NLR) is associated with poor outcomes. Based on alterations in sepsis, we hypothesized that we could observe in cirrhotic and LT patients more detailed neutrophil and lymphocyte phenotypes. To this end, along with leukocyte count, we assessed immature neutrophils, LOX-1^+^ MDSC and PD-1 and TIM-3 lymphocyte expressions in cirrhotic patients before transplantation in association with liver disease severity and during the first month after transplantation.

**Methods:** We conducted a prospective monocentric study including cirrhotic patients registered on LT waiting-list. Blood samples were collected at enrolment before LT and for 1 month post-LT. In addition to NLR, we assessed by whole blood flow cytometry the absolute count of immature neutrophils and LOX-1^+^ MDSC as well as the expressions of immune checkpoint receptors PD-1 and TIM-3 on T lymphocytes.

**Results:** We included 15 healthy volunteers (HV) and 28 patients. LT was performed for 13 patients. Pre-LT patients presented with a higher NLR compared to HV and NLR was associated with cirrhosis severity. Increased immature neutrophils and LOX-1^+^ MDSC counts were observed in the most severe patients. These alterations were mainly associated with acute decompensation of cirrhosis. PD-1 and TIM-3 expressions on T lymphocytes were not different between patients and HV. Post-LT immune alterations were dominated by a transitory but tremendous increase of NLR and immature neutrophils during the first days post-LT. Then, immune checkpoint receptors and LOX-1^+^ MDSC tended to be overexpressed by the second week after surgery.

**Conclusion:** The present study showed that NLR, immature neutrophils and LOX-1^+^ MDSC counts along with T lymphocyte count and checkpoint inhibitor expression were altered in cirrhotic patients before and after LT. These data illustrate the potential interest of immune monitoring of cirrhotic patients in the context of LT in order to better define risk of sepsis. For this purpose, larger cohorts of patients are now necessary in order to move forward a more personalised care of LT patients.

## Introduction

Liver cirrhosis defined by annular fibrosis surrounding regenerating hepatocytes is the terminal evolution of many chronic liver diseases (Anthony et al., 1977). Advanced stages of cirrhosis are characterized by portal hypertension, hepatic insufficiency and by the occurrence of progressive immune alterations known as CAID (Cirrhosis Associated Immune Dysfunction). CAID associates both systemic inflammation and features of immunosuppression as a consequence of alterations of the gut-liver axis inducing intestinal hyper-permeability and dysbiosis (Albillos et al., 2021). This leads to a continuous immune stimulation by microbial antigens and ultimately to immune cell exhaustion (Albillos et al., 2020). As a result, both innate and adaptive immune responses are dysregulated in cirrhotic patients and dramatically worsen with cirrhosis severity such as in the highest severity stage of inflammation represented by ACLF (Acute on Chronic Liver Failure) (Arvaniti et al., 2010). In this context of advanced cirrhosis, liver transplantation (LT) remains the only curative treatment. In addition to CAID, LT amplifies the profound immunosuppressive state of patients due to major surgery, immunosuppressive drugs, and intensive care unit stay). Therefore, infections constitute a major clinical issue in pre- and post-LT patients (Tranah et al., 2022). Before LT, infections in cirrhotic patients are both more frequent and more severe in association with cirrhosis severity and they can delay the access to a graft and increase mortality risk (Finkenstedt et al., 2013). After LT, infections increase morbidity and graft dysfunction (Tranah et al., 2022). Noteworthy, infections represent the major cause of death in the first year following LT in ACLF patients (Sundaram et al., 2020).

Sepsis, a life-threatening organ dysfunction caused by a dysregulated inflammatory host response to infection, shares many similarities with decompensated cirrhosis in terms of immuno-inflammatory response (Singer et al., 2016). It associates overwhelming inflammation and compensatory anti-inflammatory response that may lead to marked immunosuppression. Besides, immune dysfunction in ACLF has been described as a “sepsis-like” immune paralysis (Wasmuth et al., 2005). In sepsis, many immunological parameters have been demonstrated as prognostic marker of higher infectious rick/mortality (Venet and Monneret, 2018). Of them, due to lymphocyte apoptosis and emergency granulopoiesis (Venet et al., 2021) the neutrophil-lymphocyte ratio (NLR) is a widely described prognostic biomarker associated with poor outcomes (Rehman et al., 2020; Abensur Vuillaume et al., 2021; Lorente et al., 2022). Moreover, on neutrophil side, additional dysfunctional subsets have been described: increased immature neutrophils (i.e., CD16^low^) (Rehman et al., 2020) or occurrence of LOX-1+ myeloid derived suppressive cells (LOX-1^+^ MDSC) (Coudereau et al., 2022). On lymphocyte side, several reports revealed overexpression of immune checkpoint receptors such as PD-1 (Programmed death-1) and TIM3 (T cell immunoglobulin domain and mucin domain 3) on lymphocyte surface (Guignant et al., 2011; Boomer et al., 2012). Most importantly, in septic patients, all these parameters contribute to immunosuppression and were repeatedly reported to be associated with poor outcomes (mortality, risk of secondary infections, and longer length of ICU stay) (Venet and Monneret, 2018).

So far, although NLR has been studied in cirrhosis (Cai et al., 2017; Bernsmeier et al., 2020; Liu et al., 2021; Magalhães et al., 2021) but never after LT, further phenotyping of additional cell subsets (either neutrophils or lymphocytes) has never been conducted, especially over the pre/post-transplantation period. Explorations in the field may address the unmet clinical need in early recognition of infectious risk in cirrhotic and LT patients. Having similar NLR alterations in cirrhosis and sepsis, we hypothesize that we could observe in cirrhotic patients more detailed neutrophil and lymphocyte phenotype alterations known to be associated with immunosuppression. To this end, along with leukocyte count, we assessed immature neutrophils, LOX-1^+^ MDSC and PD-1 and TIM3 lymphocyte expression in cirrhotic patients before transplantation in association with liver disease severity and during the first month after transplantation. We aimed to better characterize immune alterations in those patients to identify putative biomarkers that may help in defining more individualized medicine.

## Materials and methods

### Subjects

Patients registered on LT waiting for decompensated cirrhosis or for cirrhosis complicated with hepatocellular carcinoma list at Lyon University Hospital (France) were prospectively enrolled. All patients were eligible to a standard immunosuppressive protocol with administration of simulect (day 0 and day 4), corticoids (at least 7 days), tacrolimus and mycophenolate mofetil. Exclusion criteria were as follows: patients requiring multi-organ transplant, patients treated with immunosuppressors (including patients with history of previous LT) and patients without underlying cirrhosis. This protocol is an ancillary study from EdMonHG study (N°ID-RCB 2019-A00954-53, CT identifier: NCT03995537).

Patients reported in this study were included from January 2022 to September 2022. Peripheral blood samples were collected once at enrolment (within 3 months before LT). Following LT, samples were collected twice a week for 1 month or until the occurrence of infection and/or acute cellular rejection. Post-LT time points were grouped as follows: day 1 to day 3 (D1-D3), day 4 to day 6 (D4-D6), day 7 to day 13 (D7-D13), day 14 to day 20 (D14-D20), day 21 to day 27 (D21-D27) and day 28 to day 31 (D28-D31). Before LT, all clinical data related to cirrhosis severity and aetiologies were collected. All relevant clinical and biological data occurring during and after transplant surgery were recorded. Acute decompensation (AD) of cirrhosis was defined by the acute development of one or more major complications of liver disease (i.e., ascites, hepatic encephalopathy, gastrointestinal haemorrhage and/or bacterial infections) (Moreau et al., 2013). ACLF stage in pre-LT patients were defined according to Moreau’s criteria (Moreau et al., 2013). Pre-LT patients were divided into two groups according to Model of End stage Liver Disease (MELD) score, a validated chronic liver disease scoring system that predicts 3-month survival on liver waiting list. A cut-off of MELD score ≥30 was chosen to identify the most severe patients. In addition, patients were stratified according to the Child-Pugh score, which is a clinico-biological scoring system used to assess prognosis of cirrhotic patients. We compared Child-Pugh A or B patients (A/B) with Child-Pugh C patients (the most severe patients).

After LT, any event of acute cellular rejection or sepsis occurrence, according to the criteria of the American Society of Transplantation (Humar et al., 2006) stopped the immune monitoring (i.e., censured forthcoming results) since they both impact immune functions by themselves. Fifteen healthy volunteers (HV) served as controls (samples coming from French Blood Establishment). The median age of HV was 38 years and 33% were male.

### Whole blood phenotyping

At each time point, in addition to leukocyte count, we assessed immature neutrophils (CD16^low^) and LOX-1^+^ MDSC (CD15^+^, CD45^dim^, LOX-1^+^ polymorphonuclear cells) percentages as described by Coudereau et al. (2022) and immune checkpoint inhibitor (PD-1 and TIM-3) expression on CD3, CD4 and CD8 T lymphocytes. Cell staining was performed on fresh whole blood sample within 4 h after sampling. We used the following antibodies: CD45-PB, CD3-APC-AF750, CD4-FITC, CD8-Kro, CD14-PB, CD16-APC from BeckmanCoulter (Brea, CA) and: PD1-APC, TIM-3-PE-Dazzle, CD15-AF700, LOX1-PE from BioLegend (San Diego, CA). Isotype control antibodies (BioLegend) were used to determine the percentages of positive cells for PD-1, TIM-3 and LOX-1. Samples were run on Navios flow cytometer (Beckman Coulter). T lymphocytes subsets’ absolute quantification was performed on Aquios flow cytometer (Beckman Coulter). Detailed protocols are presented in supplementary methods. Results were expressed as absolute counts for neutrophil subsets and T lymphocyte subsets (i.e., cells/mm3). Results were expressed as absolute cell counts for immature neutrophils and LOX-1^+^ MDSC. Immune checkpoint inhibitor expressions on T lymphocyte subsets were expressed as percentages of positive cells based on isotype controls.

### Statistics

Statistical analyses were performed with the software RStudio (2021.09.2 + 382 version). Data are presented on boxplot graph with medians, interquartile ranges and individual values. Non-parametric Mann-Whitney, Fisher’s exact test and χ2 tests were used to assess differences between groups. When appropriate, ANOVA test was used to assess differences between more than 2 independent groups. If ANOVA assumptions were not verified Kruskal–Wallis test was performed. Spearman coefficient was used to assess correlation between quantitative data. Statistical significance was assumed at *p* < 0.05. Due to relatively low number of transplanted patients, we did not perform statistical analysis after LT. Given the exploratory nature of the present observational study, no power analysis was performed.

## Results

### Patients’ characteristics

**FIGURE 1:**
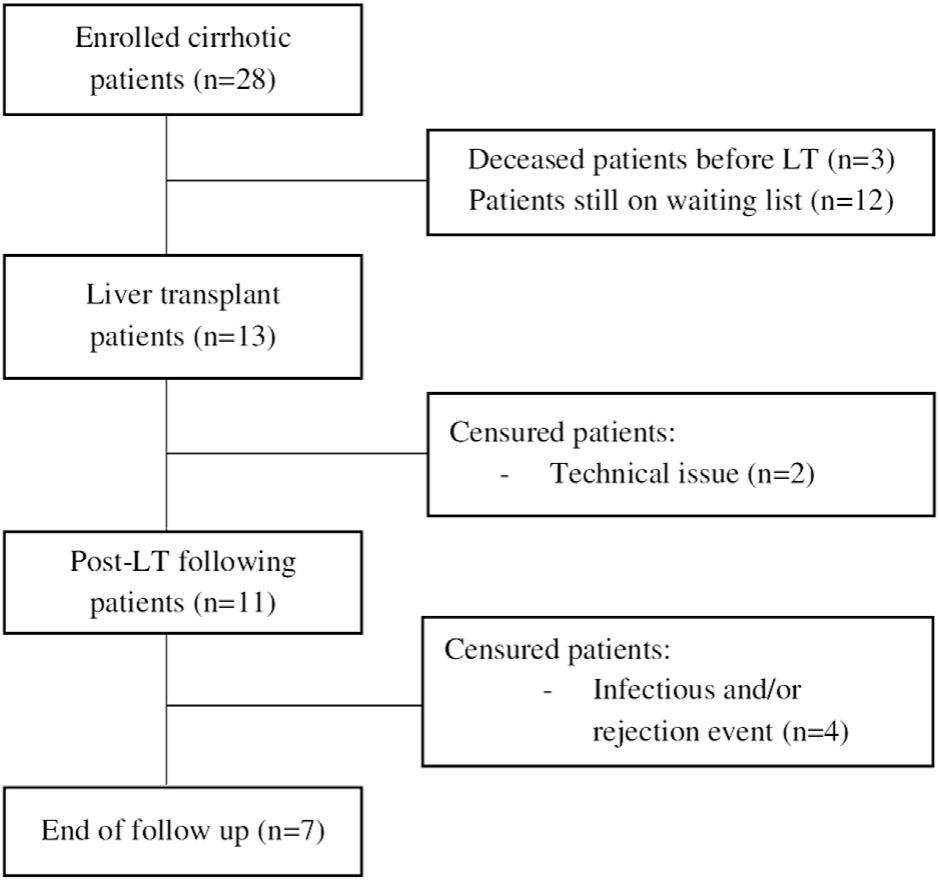
Flow chart.

During the study period, 28 cirrhotic patients were enrolled in this study. Clinical characteristics are presented in Table 1. Briefly, the median age was 58 years and 86% were male. Alcohol-related liver disease represented 53% of the cirrhosis aetiology. 7% of patients had a dysmetabolic cirrhosis and 25% had a mixed cirrhosis (5 patients had a cirrhosis related to dysmetabolic syndrome and alcohol intake and 2 patients had a cirrhosis related to HCV or HBV infection and alcohol intake). One patient had a post hepatitis C cirrhosis. The two patients with background of hepatitis C obtained a viral clearance years before inclusion. The patient with hepatitis B had a patent HBV reactivation at inclusion. 26% of patients had MELD score ≥30 (n = 8) and 43% were in AD (n = 12). Among AD patients, 83% met ACLF criteria (n = 10). All the patients with a MELD score ≥30 were in AD and met ACLF criteria. The causes of AD were infections (n = 8), acute alcoholic hepatitis (AAH) (n = 1), alcohol consumption without AAH (n = 1), HBV reactivation (n = 1) and Wilson’s disease exacerbation (n = 1). 38% of patients with a MELD score ≥30 died on waiting list (n = 3). In this cohort, 46% of patients (n = 13) underwent LT (table 2). Of them, 11 were monitored over post-LT period (2 were missing due to mistakes in protocol guidance). Seven patients completed the whole follow-up, 3 presented with sepsis, and last one presented both infection and rejection. Patient’s flow chart is presented in Figure 1. Events of infection and reject are summarised in Table 3.

**TABLE 1:** Patients characteristics of whole cohort and according to MELD score.

Patients characteristics	All patients (n = 28)	Patients with MELD <30 (n = 20)	Patients with MELD ≥30 (n = 8)	p
Demographic characteristics				
Age (years)	58 [37—68]	61.5 [48—68]	55 [37—61]	<0.01
Sex (male)	24 (86)	17 (85)	7 (88)	NS
Cirrhosis Aetiology				
Alcohol	15 (53)	10 (50)	5 (63)	NS
Dysmetabolic	2 (7)	2 (10)	0 (0)	
HCV	1 (4)	1 (5)	0 (0)	
Mixed cirrhosis				
Alcohol/dysmetabolic	5 (18)	5 (25)	0 (0)	
Alcohol/viruses	2 (7)	1 (5)	1 (13)	
Others	3 (11)	1 (5)	2 (25)	
Decompensation stages				<0.001
Compensated	8 (29)	8 (40)	0 (0)	
Chronic decompensation (CD)	8 (29)	8 (40)	0 (0)	
Acute decompensation (AD)	12 (43)	4 (20)	8 (100)	
Aetiology of AD				NS
Infection	8 (67)	4 (100)	4 (50)	
AAH	1 (8)	0 (0)	1 (13)	
HBV reactivation	1 (8)	0 (0)	1 (13)	
Wilson disease	1 (8)	0 (0)	1 (13)	
Alcohol intake	1 (8)	0 (0)	1 (13)	
Clinical parameters				
Active smokers	10 (36)	8 (40)	2 (25)	NS
Diabetes	9 (32)	9 (45)	0 (0)	0.03
HBP	12 (42)	10 (50)	2 (25)	NS
Chronic ascitis	10 (36)	7 (35)	3 (38)	NS
HE (at inclusion)	7 (25)	4 (20)	3 (38)	NS
AKF (at inclusion)	4 (14)	0 (0)	4 (50)	<0.001
Biologic markers				
Bilirubin	70 [5.8—679]	51.8 [5.8—330]	468 [71—679]	<0.001
ALP	108.5 [63—250]	113 [63—250]	69.5 [63—177]	NS
GGT	61.5 [21—273]	68 [21—273]	53 [28—246]	NS
ALT	36.5 [14—139]	33.5 [14—74]	61.5 [23—139]	0.02
AST	54 [15—286]	50.5 [15—130]	81.5 [54—286]	<0.001
Albumin	34.8 [21.5—45.7]	35.7 [22.5—45.7]	24.9 [21.5—39.4]	NS
Sodium	137 [128—142]	136.5 [130—142]	137.5 [128—140]	NS
PT	37.5 [14—100]	45.5 [26—100]	26.5 [14—52]	<0.01
INR	2.02 [1—5.5]	1.79 [1—2.99]	2.88 [1.65—5.5]	<0.01
Factor V	37 [10—123]	59 [21—123]	28.5 [10—76]	0.04
Creatinine	68 [36—275]	63.5 [36—127]	166 [41—275]	NS
Platelets (G/L)	82.5 [12—243]	108 [12—243]	64 [22—216]	NS
Hemoglobin (mg/dL)	9.5 [5.7—16.4]	10.6 [5.7—16.4]	8.7 [6.0—12.1]	0.03
CRP (mg/dL)	15.7 [0.5—50.9]	8.9 [0.5—50.9]	22,9 [19.6—44.7]	0.008
Pronostic scores				
MELD score	24 [6—40]	18 [6—27]	35 [30—40]	<0.001
Child-Pugh score	10 [5—14]	8 [5—13]	11 [10—14]	<0.01
Child-Pugh C	16 (57)	8 (40)	8 (100)	<0.01
SOFA score	4.5 [0—15]	3 [0—10]	8 [6—15]	<0.001
ACLF	10 (36)	2 (10)	8 (100)	<0.001
Immunologic parameters				
Neutrophils (G/L)	4.1 [1.6—20.9]	3.4 [1.6—8.5]	4.9 [2.8—20.9]	0.008
Monocytes (G/L)	0.65 [0.33–1.89]	0.72 [0.33—1.48]	0.59 [0.35—1.89]	NS
T lymphocytes (cells/μL)	507 [79—1,479]	737 [79–1,479]	372 [204—1,395]	0.03
NLR	3.1 [1.6—40.2]	2.4 [1.6—8.2]	8.9 [4.5—40.2]	<0.001
Death on waiting list	3 (11)	0 (0)	3 (38)	NS

Quantitative data are presented as medians with minimum and maximum value within square brackets [min–max]. Qualitative data are presented as numbers of cases and percentage among the total population or subpopulation in brackets (%). Prognostic scores and immunologic parameters were calculated the day of patients’ inclusion. *p*-values were calculated using Mann-Whitney, Fisher and χ2 tests when appropriate. AAH (acute alcoholic hepatitis). ACLF (acute on chronic liver failure). AD (acute decompensation of cirrhosis). AKF (acute kidney failure). ALP (alkaline phosphatase). ALT (alanine aminotransferase). AST (alanine aminotransferase). CD (chronic decompensation of cirrhosis). CRP (c-reactive protein). MELD (Model of End Stage Liver Disease). NLR (neutrophils to lymphocytes ratio). HBP (high blood pressure). HCV (hepatitis C virus). HE (hepatic encephalopathy). GGT (gamma glutamyl transferase). INR (international standardization ratio). PT (prothrombin time). SOFA (Sequential Organ Failure Assessment).

**TABLE 2:** Characteristics of transplanted patients.

Patients characteristics	Transplant patients (n = 13)
Decompensation stages	
Compensated	3 (23)
Chronic decompensation (CD)	3 (23)
Acute decompensation (AD)	7 (53)
Pronostic scores	
MELD score ≥30	4 (30)
Child-Pugh C	9 (69)
ACLF	6 (46)
Liver surgery	
Surgery time (minutes)	450 [248—525]
Cold ischaemia (minutes)	420 [278—560]
Red cells transfusion	2 [0—10]
Post-transplant outcomes (during the first month post LT)	
Intensive care length of stay (days)	6 [4—79]
Total duration of vasopressors (days)	0 [0—8]
Total duration of MV (days)	0 [0—29]
Surgical revision	4 (31)
Graft dysfunction at day 7^*^	6 (46)
Infectious event	5 (38)
Reject	1 (8)
One month survival	13 (100)

**TABLE 3:** Infectious and graft rejection outcomes.

Patients	MELD score	ACLF	Clinical events	Identified germ	Post-transplant days	Intensive care unit stay	One month survival
**1**	19	No	Pneumoniae and acute cellular rejection	*Klebsiella pneumoniae*	D5 (infection) D6 (reject)	7	yes
**2**	30	No	Peritonitis	No	D12	72	yes
**3**	27	No	Pneumoniae	*Pseudomonas aeruginosa*	D10	68	yes
**4**	36	Yes	Infectious pleuritis	*Enterococcus* faecium	D17	still in ICU at Ms submission (i.e., 85 days)	yes

## Pre-transplantation results

### Total neutrophil count and neutrophil-lymphocyte ratio

**FIGURE 2:**
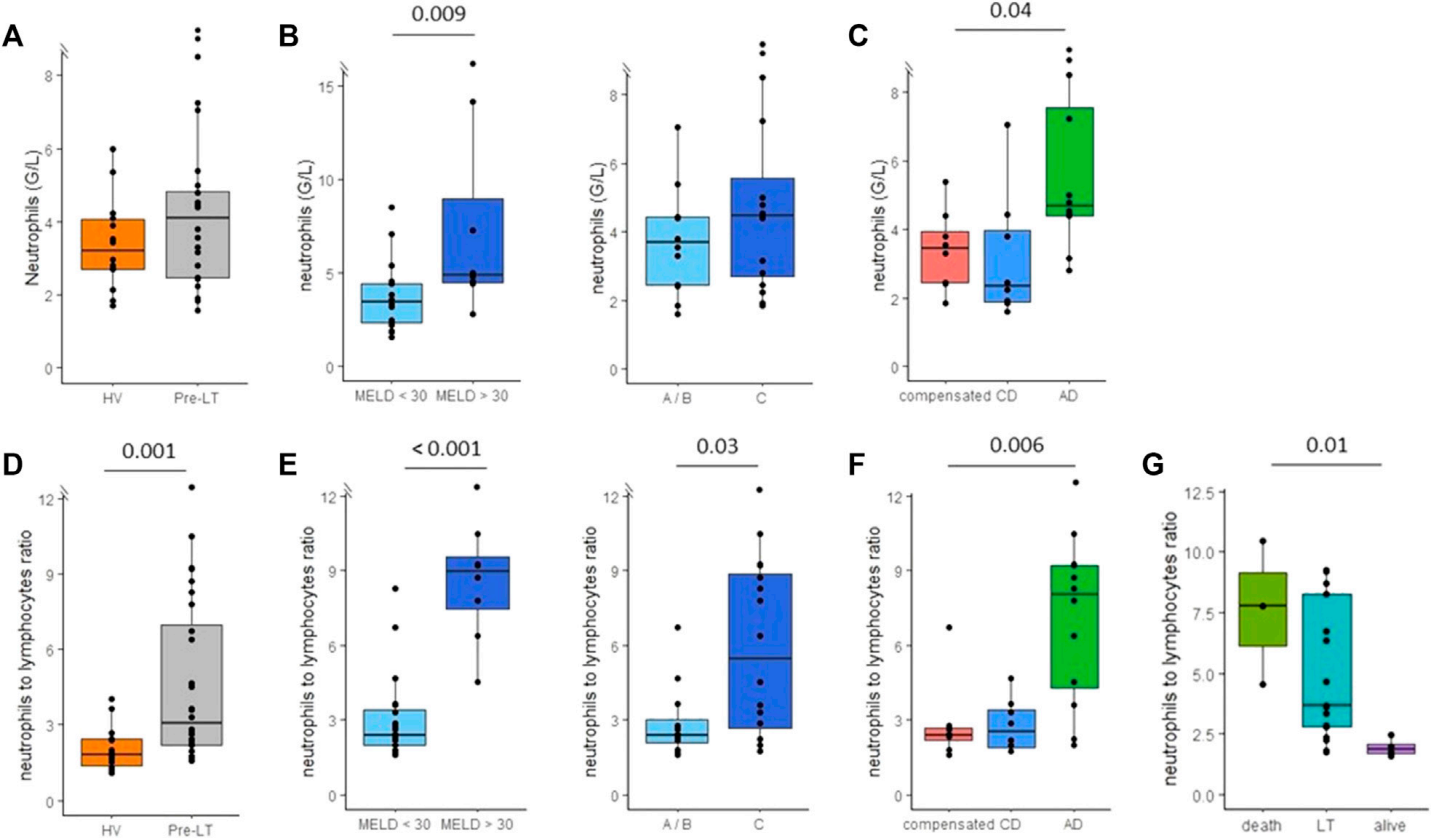
Monitoring of neutrophils count and neutrophils to lymphocytes ratio (NLR) in peripheral blood of pre-transplant patients. **(A)** Neutrophils count in healthy volunteers (HV, *n* = 15) and pre-transplant patients (pre-LT, *n* = 28). **(B)** Neutrophils count in patients with MELD score < 30 (*n* = 20) or with MELD score ≥ 30 (*n* = 8) and in patients with Child-Pugh score A or B (A/B, *n* = 12) or with Child-Pugh score C (*n* = 16). **(C)** Neutrophils count in patients with compensated cirrhosis (*n* = 8), chronic decompensated cirrhosis (CD, *n* = 8) and acute decompensated cirrhosis (AD, with *n* = 10 or without ACLF *n* = 2). **(D)** NLR in healthy volunteers (HV) and pre-transplant patients (pre-LT). **(E)** NLR in patients with or without MELD score < 30 and in patients with Child-Pugh A/B or C. **(F)** NLR in patients with compensated, chronic decompensated and acute decompensated cirrhosis. **(G)** NLR according to three months evolution post inclusion. The nonparametric Wilcoxon test was used to assess differences between patients and HV and between patients’ subgroups determined by MELD and Child-Pugh scores. ANOVA or Kruskal-Wallis tests were used to assess differences between more than 2 independent groups.

Neutrophil count was not significantly different in pre-LT patients (whole cohort) in comparison to HV (Figure 2A). Nevertheless, increased neutrophils were associated with more severe cases according to MELD score (Figure 2B) and were associated with decompensation stages of cirrhosis (Figure 2C). NLR was significantly increased in pre-LT patients in comparison to HV (Figure 2D). Importantly, NLR was higher in patients with MELD score ≥30 and Child-Pugh score C (Figure 2E). Moreover, NLR was significantly associated with decompensation stages of cirrhosis as it was predominantly increased in AD patients (Figure 2F). There was a positive correlation between NLR and MELD score (r = 0.7; *p* < 0.001) and between NLR and CRP (r = 0.74; *p* = 0.001). Interestingly, NLR was significantly associated with patients’ survival 3 months after inclusion (Figure 2G). There was no transplant free survival in patients with NLR >4. The cause of death was multiple organ failure syndrome secondary to uncontrolled infection for the three patients who died on waiting list.

### Neutrophil subsets

**FIGURE 3:**
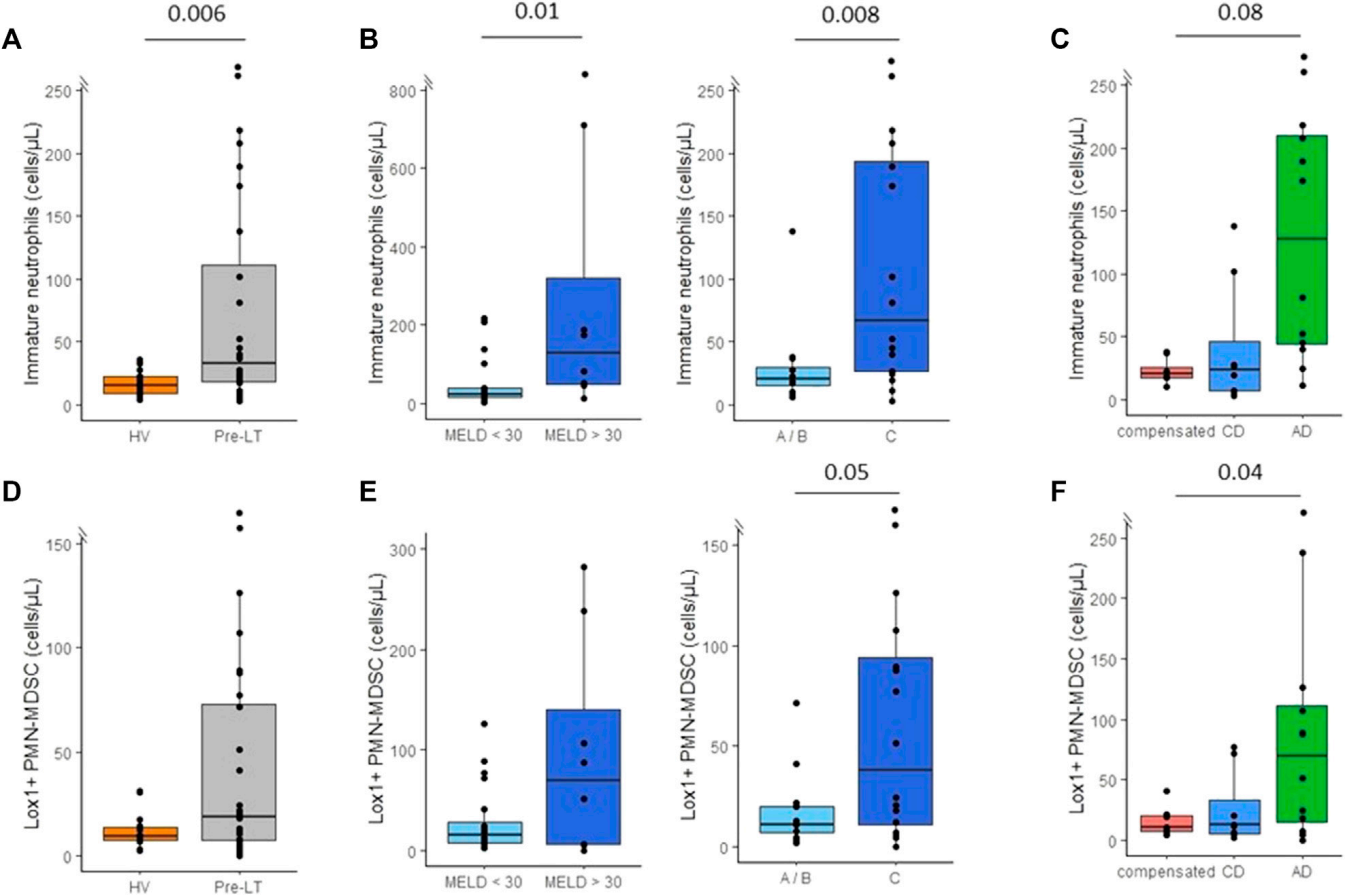
Monitoring of immatures neutrophils (CD16^low^) and lectine-type oxidized LDL receptor 1 polymorphonuclear myeloid-derived suppressor cells (LOX1+ PMN-MDSC) in peripheral blood of pre-transplant patients. **(A)** Immature neutrophils count in healthy volunteers (HV, *n* = 15) and pre-transplant patients (pre-LT, *n* = 28). **(B)** Immature neutrophils count in patients with a MELD score < 30 (*n* = 20) or with a MELD score ≥ 30 (*n* = 8) and in patients with Child-Pugh score A or B (A/B, n=12) or with Child-Pugh score C (n=16). (C) Immature neutrophils count in patients with compensated cirrhosis (*n* = 8), chronic decompensated cirrhosis (CD, *n* = 8) and acute decompensated cirrhosis (AD, with *n* = 10 or without ACLF *n* = 2). **(D)** Number of LOX1+ MDSC in healthy volunteers (HV) and pre-transplant patients (pre-LT). **(E)** Number of LOX1+ MDSC in patients with or without MELD score < 30 and in patients with Child-Pugh A/B or C. **(F)** Number of LOX1+ MDSC in patients with compensated, chronic decompensated, and acute decompensated cirrhosis. The nonparametric Wilcoxon test was used to assess differences between patients and HV and between patients’ subgroups determined by MELD and Child-Pugh score. Kruskal-Wallis test was used to assess differences between more than 2 independent groups.

Immature CD16^low^ neutrophil counts were significantly increased in pre-LT patients in comparison with HV (Figure 3A). Increased immature neutrophils count was associated with cirrhosis severity according to MELD and Child-Pugh scores (Figure 3B). Moreover, AD patients tended to show increased immature neutrophils count in comparison with compensated and CD patients (Figure 3C). In addition, we found a positive and significant correlation between immature neutrophil counts and CRP (r = 0.60, *p* = 0.016) and MELD score (r = 0.56, *p* = 0.0039). Although clearly elevated in some patients, LOX-1^+^ MDSC counts were not significantly different between patients and HV (Figure 3D). Regarding association with pre-LT severity, solely AD patients presented with significantly elevated values (Figure 3F). Importantly, immature neutrophils and LOX-1^+^ MDSC counts were significantly correlated to NLR (r = 0.57, *p* = 0.002; and r = 0.4, *p* = 0.034 respectively). Noteworthy, immature neutrophils and LOX-1^+^ MDSC counts were not increased neither in patients with active hepatocellular carcinoma (n = 4) nor with patients transplanted for hepatocellular carcinoma (n = 10) (data not shown).

### T lymphocyte counts

**FIGURE 4:**
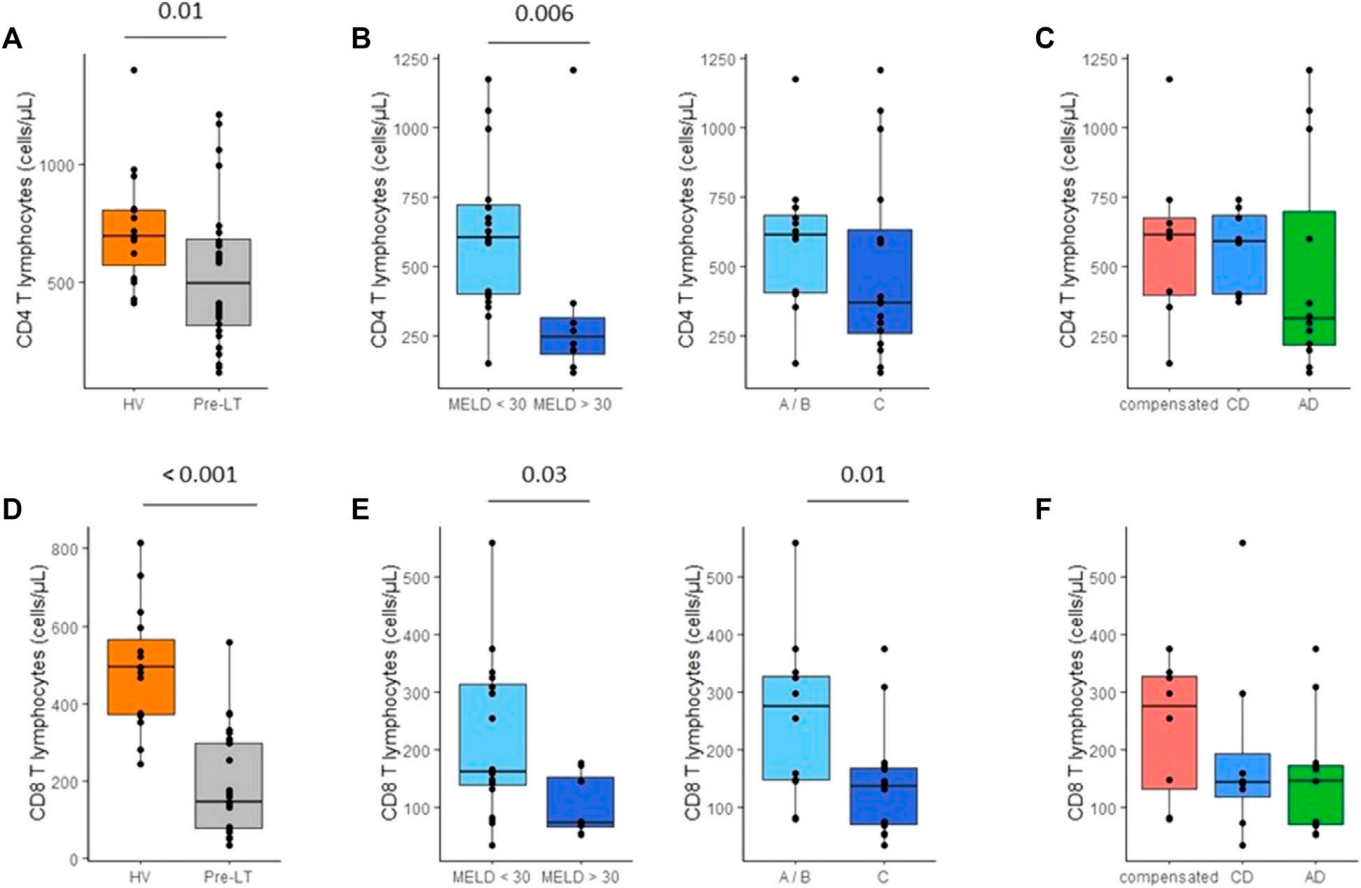
Monitoring of CD4+ and CD8+ T cell counts in peripheral blood of pre-transplant patients. **(A)** CD4+ T lymphocytes count in healthy volunteers (HV, *n* = 15) and pre-transplant patients (pre-LT, *n* = 28). **(B)** CD4+ T lymphocytes count in patients with a MELD score < 30 (*n* = 20) or with a MELD score ≥ 30 (*n* = 8) and in patients with Child-Pugh score A or B (A/B, *n* = 12) or with Child-Pugh score C (*n* = 16). **(C)** CD4= T lymphocytes count in patients with compensated cirrhosis (*n* = 8), chronic decompensated cirrhosis (CD, *n* = 8). **(D)** CD8+ T lymphocytes count in healthy volunteers (HV) and pre-transplant patients (pre-LT). **(E)** CD8+ T lymphocytes count in patients with or without MELD score < 30 and in patients with Child-Pugh A/B or C. **(F)** CD8+ T lymphocytes count in patients with compensated, chronic decompensated, and acute decompensated cirrhosis. The nonparametric Wilcoxon test was used to assess differences between patients and HV and between patients’ subgroups determined by MELD and Child-Pugh score.

We observed a profound T lymphopenia in cirrhotic patients in comparison to HV. This affected both CD4^+^ (median: 496 CD4^+^ cells/mm^3,^ Figure 4A) and CD8^+^ (median: 148 CD8^+^ cells/mm^3,^ Figure 4D) T lymphocyte subsets in pre-LT patients. Lymphopenia was significantly more important in patients with MELD score ≥30 compared to patients with MELD score <30 (Figures 4B, E). Interestingly, CD8^+^ T cells count was significantly decreased in compensated patients in comparison to HV (*p* = 0.002). Moreover, lymphopenia tented to accentuate during decompensated stages of cirrhosis (Figures 4C, F). CD3^+^ T cells count was negatively correlated to CRP (r = −0.73; *p* = 0.002).

### Immune checkpoint inhibitor expressions on T lymphocyte subsets

**FIGURE 5:**
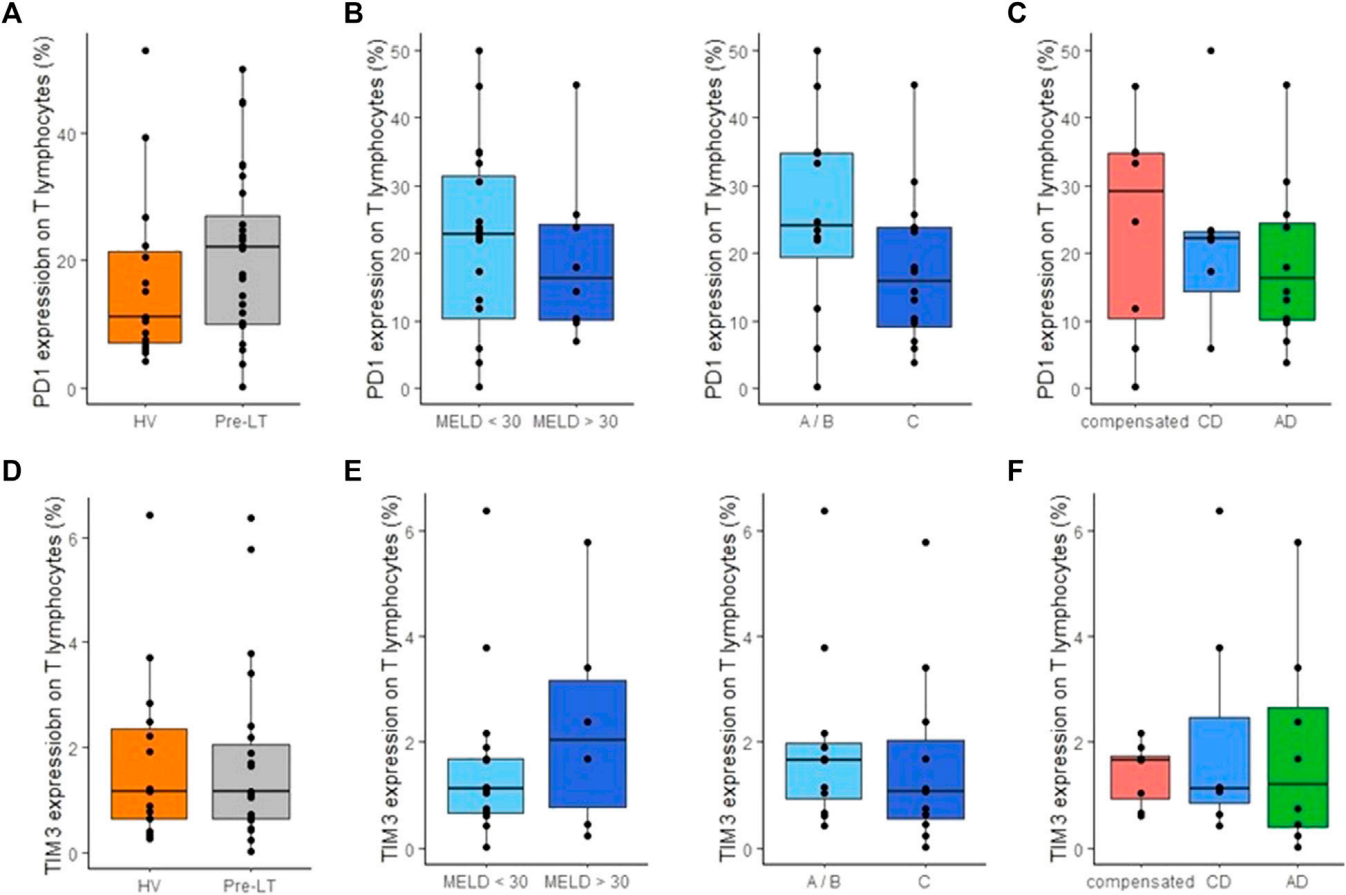
Monitoring of PD-1 and TIM3 expression on T cells in peripheral blood of pre-transplant patients. **(A)** Percentage of PD-1 expression on CD3+ T lymphocytes in healthy volunteers (HV, *n* = 15) and pre-transplant patients (pre-LT, *n* = 28). **(B)** Percentage of PD-1 expression on CD3+ T lymphocytes in patients with a MELD score < 30 (*n* = 20) or with a MELD score ≥ 30 (*n* = 8) and in patients with Child-Pugh score A or B (A/B, *n* = 12) or with Child-Pugh score C (*n* = 16). **(C)** Percentage of PD-1 expression on CD3+ T lymphocytes in patients with compensated cirrhosis (*n* = 8), chronic decompensated cirrhosis (CD, *n* = 8) and acute decompensated cirrhosis (AD, with *n* = 10 or without ACLF *n* = 2). **(D)** Percentage of TIM3 expression on CD3+ T lymphocytes in healthy volunteers (HV) and pre-transplant patients (pre-LT). **(E)** Percentage of TIM3 expression on CD3= T lymphocytes in patients with compensated, chronic decompensated, and acute decompensated cirrhosis. **(F)** Percentage of PD-1 expression on CD3+ T lymphocytes in patients with or without a MELD score < 30 and in patients with Child-Pugh A/B or C. The nonparametric Wilcoxon test was used to assess variations between patients and HV and between patients’ subgroups determined by MELD and Child-Pugh score. Kruskal-Wallis test was used to assess differences between more than 2 independent groups.

PD-1 and TIM3 expressions on CD3^+^ T lymphocytes were not different between HV and pre-LT patients (Figures 5A, D). Overall, PD-1 and TIM3 expressions were not associated with cirrhosis severity according to MELD and Child-Pugh scores (Figures 5B, E) or with decompensation stages of cirrhosis (Figures 5C, F). These results were similar on CD8^+^ and CD4^+^ T cells (data not shown). Importantly, as alcohol is able to induce PD-1 and TIM3 expressions *in vitro* (Markwick et al., 2015), we verified that immune checkpoint receptors were not differently expressed in alcohol consumer patients (n = 5) compared non-alcoholic and weaned patients (n = 23, data not shown). Moreover, as immune checkpoint receptors might be overexpressed in cancer, we addressed this aspect but noticed that PD-1 and TIM3 were not differently expressed in patients with active hepatocellular carcinoma (n = 4). In addition, there were no differences between patients enrolled on waiting list for hepatocellular carcinoma (n = 10) and patients without medical history of hepatocellular carcinoma (n = 18, data not shown).

## Post-transplant results

### Total neutrophil count, neutrophil-lymphocyte ratio and neutrophil subsets

**FIGURE 6:**
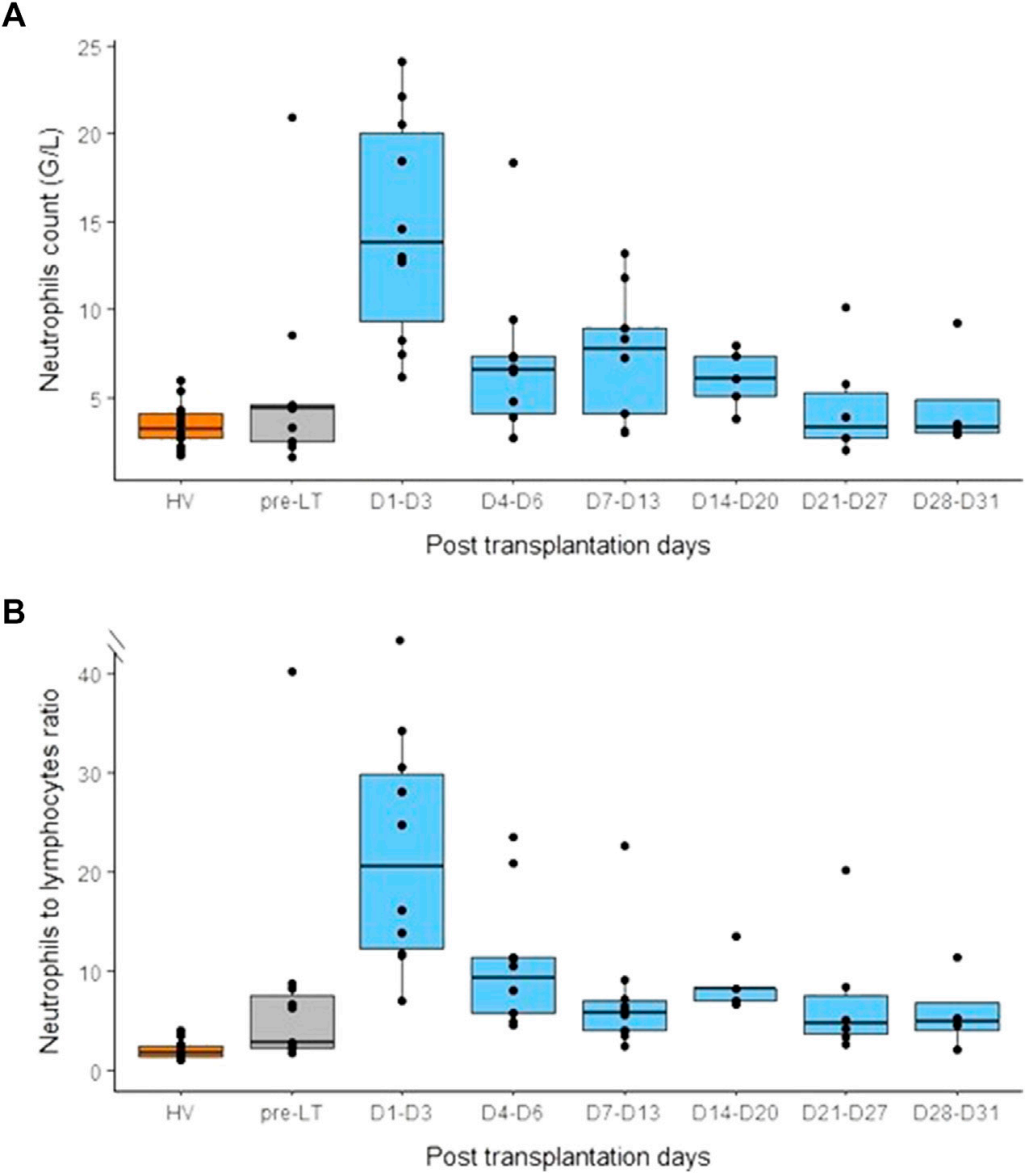
Monitoring of neutrophils count and neutrophils to lymphocytes ratio (NLR) before and after liver transplantation. **(A)** Neutrophils count in healthy volunteers (HV, *n* = 15), pre-transplant patients (pre-LT, *n* = 11) and after liver transplantation at different time points (day 1 to day 3, *n* = 10; day 4 to day 6, *n* = 10; day 7 to day 13, *n* = 10; day 14 to day 20, *n* = 5; day 21 to day 27, *n* = 6; day 28 to day 31, *n* = 4). **(B)** NLR in healthy volunteers (HV), pre-transplant patients (pre-LT) and following transplantation at different time points. Pre-transplant data only concern patients that benefited from transplantation.

**FIGURE 7:**
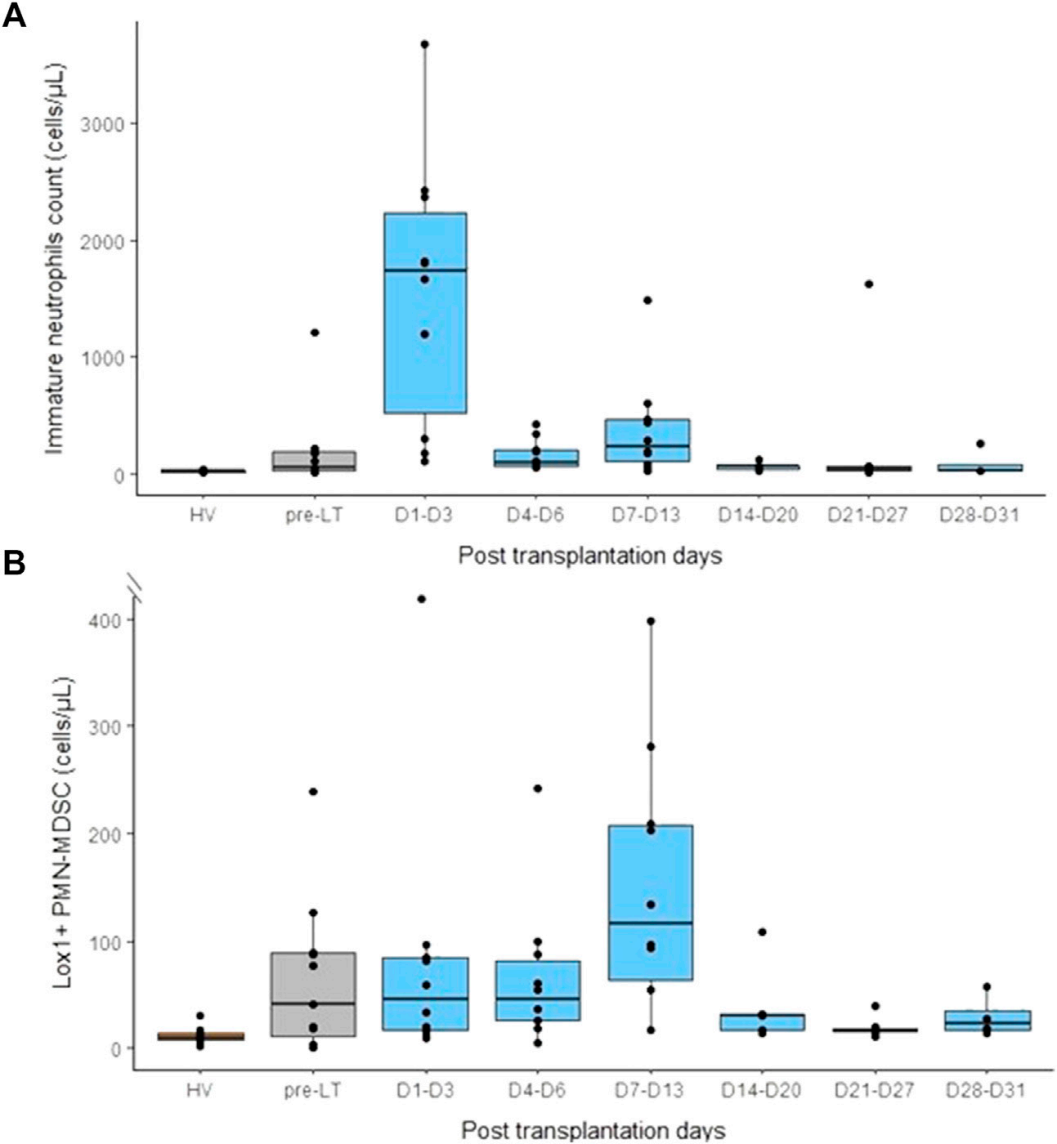
Monitoring of immature neutrophils (CD16low) and lectine-type oxidized LDL receptor 1 polymorphonuclear myeloid-derived suppressor cells (LOX1+ PMN-MDSC) in peripheral blood before and after liver transplantation. **(A)** Immature neutrophils count in healthy volunteers (HV, *n* = 15), pre-transplant patients (pre-LT, *n* = 11) and after liver transplantation at different time points (day 1 to day 3, *n* = 10; day 4 to day 6, *n* = 10; day 7 to day 13, *n* = 10; day 14 to day 20, *n* = 5; day 21 to day 27, *n* = 6; day 28 to day 31, *n* = 4). **(B)** Number of Lox1+ PMN-MDSC in healthy volunteers (HV), pre-transplant patients (pre-LT) and following transplantation at different time points. Pre-transplant data only concern patients that benefited from transplantation.

After LT, we observed a tremendous increase of neutrophils count at D1-D3 post-LT. Then, neutrophils count decreased and reached pre-LT values during the third week post-LT (Figure 6A). In accordance, we observed an important rise of NLR at D1-D3 following LT (Figure 6B). However, this elevation was transitory and decreased at D4-D6 post-LT and remained stable until 1-month post-LT. However, throughout this follow-up, NLR remained higher than that from HV controls. According to total neutrophil count, immature neutrophils count peaked at D1-D3 after LT and then returned to pre-LT values at D4-D6 (Figure 7A). At the end of follow-up, immature neutrophils count remained slightly higher (median: 37 cells/mm^3^) than HV value (median: 15 cells/mm^3^). In contrast, LOX-1^+^ MDSC count presented with a different kinetic. LOX-1^+^ MDSC count remained stable during the first week after LT (Figure 7B) but reached a maximum during the second week post-LT (D7-D13). This elevation was transitory as LOX-1^+^ MDSC rapidly went back down to low values (median: 20 cells/mm^3^) similar to those observed in HV controls (median: 9 cells/mm^3^ in HV).

### T lymphocyte counts

**FIGURE 8:**
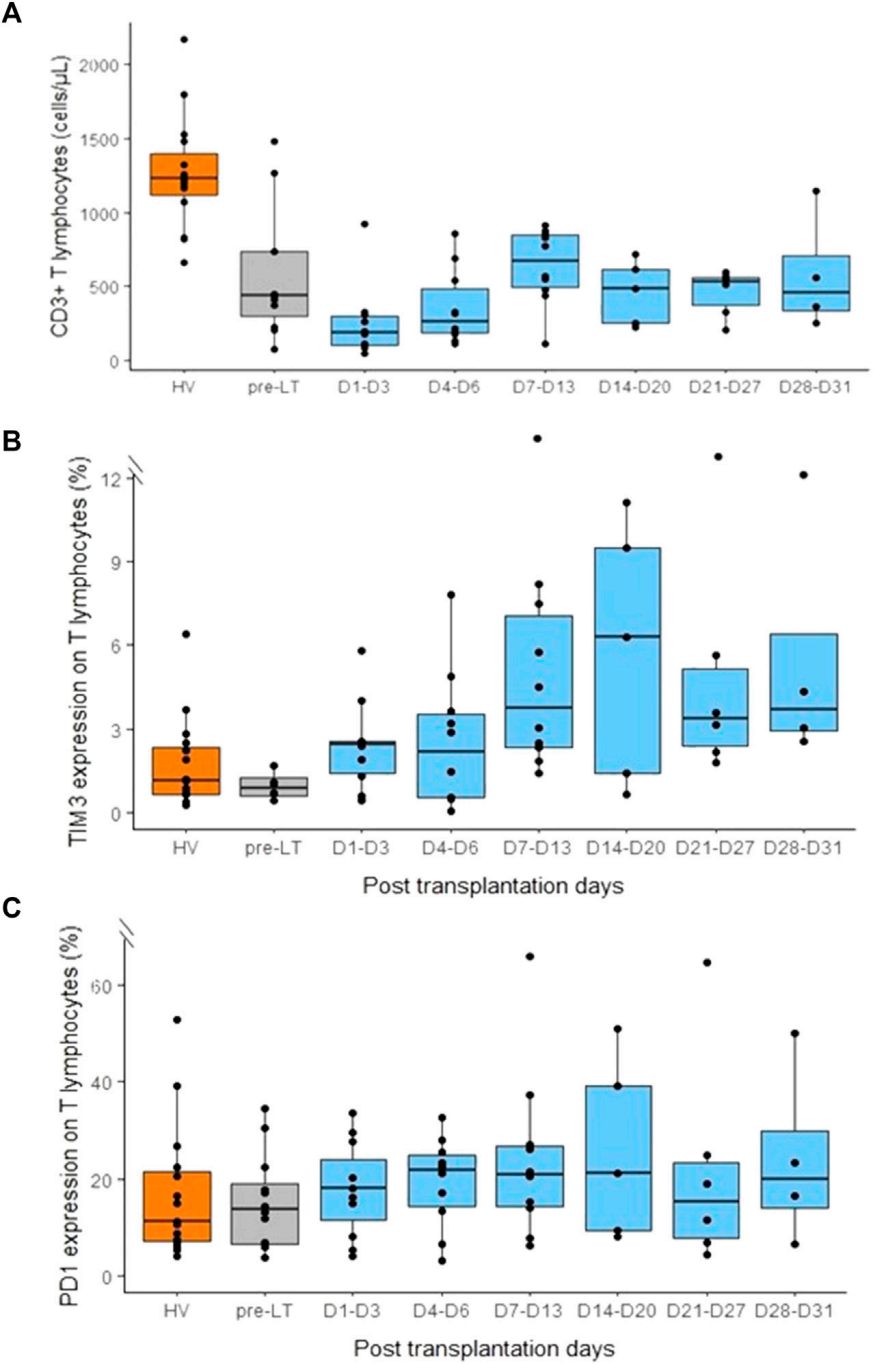
Monitoring of CD3+ T lymphocytes count and PD-1 and TIM3 expression on CD3+ T lymphocytes in peripheral blood before and after liver transplantation. **(A)** CD3+ T lymphocytes count in healthy volunteers (HV, *n* = 15), pre-transplant patients (pre-LT, *n* = 11) and following transplantation at different time points (day 1 to day 3, *n* = 10; day 4 to day 6, *n* = 10; day 7 to day 13, *n* = 10; day 14 to day 20, *n* = 5; day 21 to day 27, *n* = 6; day 28 to day 31, *n* = 4). **(B)** Percentage of TIM3 expression on CD3+ T lymphocytes in healthy volunteers (HV), pre-transplant patients (pre-LT) and following transplantation at different time points. **(C)** Percentage of PD-1 expression on CD3+ T lymphocytes in healthy volunteers (HV), pre-transplant patients (pre-LT) and following transplantation at different time points at different time points. Pre-transplant data only concern patients that benefited from transplantation.

Despite being already low before LT, lymphopenia amplified after transplantation (Figure 8A). Nadir was observed at D1-D3 post-LT. This profoundly affected all T cells subsets (medians as follows: CD3^+^ T cells: 192 cells/mm^3^, CD4^+^ T cells:131 cells/mm^3^, CD8^+^ T cells: 50 cells/mm^3^). Thereafter, T lymphocytes increased at levels similar to pre-LT values during the second week post-surgery. However, at the end of follow-up, patients still presented with marked lymphopenia (Figure 8A). In parallel, we observed a progressive over expression of both TIM-3 and PD1 checkpoint inhibitor expressions on circulating T lymphocytes, TIM3 expression reached a maximum around 2–3 weeks post-LT and then remained stable (Figure 8B). Even if it was less clear, PD-1 tended to follow same pattern of expression (Figure 8C). Similar results were observed on both CD4^+^ and CD8^+^ T lymphocytes (data not shown).

## Discussion

To the best of our knowledge, this preliminary study is the first to present a detailed neutrophils and T lymphocytes immune phenotyping overtime in cirrhotic patients before and after liver transplantation. These results provide valuable additional information and markers (LOX-1, TIM-3, PD-1) to complete previous results obtained in cirrhotic patients solely based on NLR.

NLR is believed to be associated with cirrhosis severity and mortality. Cai et al*.* reported that this parameter was an independent predictors of hospital-acquired bacterial infections in decompensated cirrhosis (Cai et al., 2017). They also demonstrated that cirrhotic patients presenting with NLR superior or equal to 4.33 had a significantly lower survival. Others studies reported that NLR was associated with mortality in cirrhosis, both in patients with MELD score < 20 (Kalra et al., 2017) and in ACLF patients (Bernsmeier et al., 2020). The present results thus confirmed those previous findings. This composite biomarker reflects the balance between granulopoiesis induced by inflammation and lymphopenia. Whereas massive rise in neutrophils occurred in the most severe cirrhotic patients (i.e., at a time of tremendous inflammation), lymphopenia seems to be an earlier event in cirrhosis pathophysiology as it appeared in patients even at compensated stage of cirrhosis. Defect of thymopoiesis and activation-driven cell-death induced by bacterial translocation have been demonstrated to sustain this lymphopenic process (Lario et al., 2013). We extended these results by showing that mostly immature neutrophils and to a lower extent immunosuppressive LOX-1^+^ MDSC contributed to neutrophil rise before LT. This suggests that neutrophil and NLR rise before LT was mainly due to massive inflammatory response and emergency granulopoiesis (including immature cells) in ACLF patients. In contrast, MDSC, usually released in a more chronic manner are less elevated. This may explain why LOX-1^+^ MDSC are less correlated to severity than neutrophils (and subsequently NLR) and immature neutrophils. Overall, the present neutrophil results completed previous studies reporting on neutrophil dysfunction in cirrhotic patients including alterations of migration, oxidative burst and phagocytic capacity (Fiuza et al., 2000; Panasiuk et al., 2005; Tritto et al., 2011). Two studies also described reduced CD16 expression on neutrophils (Taylor et al., 2014; Markwick et al., 2015) which characterizes immature neutrophils, cells known to be less efficient in opsonisation and bacteria lysis (Drifte et al., 2013).

Consequently, as observed in sepsis, the most severe cirrhotic patients with marked neutrophil phenotypic may be at higher risk of infection. In line, we observed that patients who died due to sepsis occurrence before LT presented with significantly higher NLR compared with patients who survived. In addition, we may hypothesize a role for LOX-1^+^ MDSC. Indeed, MDSC are immature neutrophils with immunosuppressive properties as they are potent repressors of T-cell response (Gabrilovich, 2017). They expand under pathological conditions associated with acute or chronic inflammation such as sepsis (Schrijver et al., 2019), cancers (Cassetta et al., 2020), or chronic infections (Pallett et al., 2015). In these contexts, the presence of PMN-MDSC respectively promoted nosocomial infections, cancer progression and persistent viral infections. In the present work, we focused on LOX-1^+^ MDSC since LOX-1 is the sole marker of granulocytic MDSC measurable in whole blood (Condamine et al., 2016; Coudereau et al., 2022). Thus, we likely underestimated the total number of MDSC. In hepatology, only one study reported of granulocytic MDSC in alcohol cirrhosis, especially in Child-Pugh B and C patients (Gao et al., 2019). In agreement, the present results showed increased LOX-1^+^ MDSC in Child-Pugh C patients. More studies are required to assess the potential role of MDSC in the pathophysiology of cirrhosis associated immune suppression.

Immune checkpoint receptors are co-inhibitory molecules expressed on immune cells that downregulate the immune response in order to promote homeostasis after immune activation. Engagement of PD-1 and TIM3 pathways on T lymphocytes leads to the inhibition of the second signal of T cell activation. High and sustained expression of the co-inhibitory molecules during persistent antigen stimulation has been shown to promote immune cells exhaustion in cancer, sepsis (Rienzo et al., 2022) and chronic hepatitis B and C (Osuch et al., 2020; Li et al., 2022). Several studies described a slight increase in PD-1 and/or TIM-3 lymphocyte expressions in acute alcoholic hepatitis/cirrhosis (Markwick et al., 2015; Lebossé et al., 2019; Riva et al., 2021; Fadriquela et al., 2022). However, in the present work, PD-1 and TIM3 expressions on T lymphocytes were not significantly different between HV and pre-LT patients and were not associated with cirrhosis severity according to MELD and Child-Pugh scores or with decompensation stages of cirrhosis. Taken together, before LT, results indicated that out of viral induced cirrhosis, infectious risk in cirrhotic patients would be more induced by immature/suppressive neutrophil subsets and profound lymphopenia rather than by increased immune checkpoint inhibitors expressions.

Regarding post-LT results, the immediate augmentation of NLR after LT is most likely the sum of multiple causes mixing both inflammatory signals and accentuated lymphopenia induced by immunosuppressant regimen, surgery, ischemia-reperfusion injury and per operative bleeding. This point needs further explorations including a larger number of patients in order to perform multiparametric analyses. As immature neutrophil count rapidly decreased after LT, it most likely does not participate to post-LT infection risk. Interestingly, LOX-1^+^ MDSC count increased 1 week after surgery. Condamine et al. revealed that these cells accumulated as the result of two groups of signals: those promoting myelopoiesis (mainly by inflammatory cytokines) and suppressive signals as occurring after transplantation (Condamine et al., 2016). In addition, as MDSC have a role in tissue repair, we may hypothesize that hepatic recruitment of these cells may contribute to counteract liver damage due to ischemia-reperfusion injury. Further exploration would be of utmost interest to associate these observations with liver dysfunction/rejection after transplantation. Not surprisingly, lymphopenia worsened days after transplantation and remained at low values throughout follow-up. Most importantly, we observed a progressive over expression of checkpoint inhibitor expressions on both CD4^+^ and CD8^+^ T cells. TIM3 expression reached a maximum around 2–3 weeks post-LT and then remained stable. In line, Mysore et al. showed that patients who developed infection during the first year post-LT had elevated co-expressions of PD-1 and TIM3 on T lymphocytes 30 days after LT (Mysore et al., 2018). Accordingly, another study revealed that PD-1 expression on CMV-specific CD8 T cells was elevated preceding CMV reactivation in LT patients (La Rosa et al., 2008). One the opposite side, checkpoint inhibitors might also contribute to immune tolerance in order to prevent graft rejection (Gong et al., 2017). Noteworthy, we noticed that during post-LT follow-up, LOX-1^+^ MDSC count and TIM-3 expression tended to peak at the same time (around 2 weeks after LT). One may hypothesize a common inducer for both mechanisms which remained to be investigated. Overall, the current preliminary data deserve further evaluations as they may provide novel understanding of immunosuppression occurring after LT.

Although the present study presents novelties regarding NLR by concomitantly assessing neutrophil (CD16^low^, LOX1^+^) and T lymphocyte (PD-1, TIM-3) subsets before and after transplantation, we acknowledge some limitations of this study. First, as a preliminary study, the number of included patients was low, especially in post-transplant period which did not allow us to associate immune parameters with clinical events after LT (sepsis, rejection). Second, only one single sample was performed pre-LT sample whereas elapsed time until transplantation was heterogeneous. This aspect should be better controlled in forthcomings studies. Lastly, along with cell count and checkpoint inhibitor expression, T cell and neutrophil functionality testing was not performed but may contribute to better understanding of post-LT immunosuppression.

In conclusion, the present study showed that NLR, immature neutrophils and LOX-1^+^ MDSC counts along with T lymphocyte count and checkpoint inhibitor expression were altered in cirrhotic patients before and after LT. These data illustrate the potential interest of immune monitoring of cirrhotic patients in the context of LT in order to better define risk of sepsis or rejection. For this purpose, larger cohorts of patients, including phenotypic and functional testing, are now necessary in order to move forward a more personalised care of LT patients.

## Data Availability

The original contributions presented in the study are included in the article/Supplementary Material, further inquiries can be directed to the corresponding author.

## Abbreviations

ACLF, Acute on Chronic Liver Failure; CAID, Cirrhosis Associated Immune Dysfunction; HV, Healthy Volunteers; Lox1, Lectine-type oxidized LDL receptor 1; LT, Liver Transplantation; MELD, Model of End stage Liver Disease; PD-1, Programmed Death 1; PMN-MDSC, Polymorphonuclear Myeloid Derived Suppressor Cells; TIM3, T cell immunoglobulin and mucin domain 3.
