# Supplementary material for: Assessment of neutrophil subsets and immune checkpoint inhibitor expressions on T lymphocytes in liver transplantation: A preliminary study beyond the neutrophil-lymphocyte ratio
Source: Front Physiol. 2023 Mar 30;14:1095723. doi: 10.3389/fphys.2023.1095723 (PMC10097891; doi:10.3389/fphys.2023.1095723)
Supplement: Supplementary file 1 [file Table1.DOCX]

**Supplementary material**

Immature neutrophils and LOX1^+^ MDSC expression

A total of 100μL of whole blood (EDTA) was labelled with antibodies CD45-Kro/CD16-PB/CD10-PE-CY7/CD15-AF700/CRTH2-APC and Lox1-PE or isotype controls IgG2aκ-PE and incubated for 15 min at room temperature in the dark. Cells were then incubated with 1mL of lysing solution (Versalye) for 10 min at room temperature in the dark. Then, they were washed with 1mL of PBS solution and incubated for 10 min at room temperature in the dark. Cells were centrifugated and supernatant were then removed. 500 μL of PBS solution were added before being analyzed by flow cytometer.

Expression of PD-1 and TIM3 on T lymphocyte cells

A total of 100μL of whole blood (EDTA) was labelled with antibodies CD45-PB/CD3-APC-AF750/CD4-FITC/CD8-Kro and TIM3-PE-Dazzle/PD1-APC or isotype controls IgG1κ-PE-Dazzle/IgG1κ-APC/ and incubated for 15 min at room temperature in the dark. Cells were then incubated with 500 μL of lysing solution (Optilyse) for 10 min at room temperature in the dark. Then, they were washed with 1mL of PBS solution and incubated for 10 min at room temperature in the dark. Cells were centrifugated and supernatant were then removed. 300 μL of PBS solution were added before being analyzed by flow cytometer.
